# On the same page? A qualitative study protocol on collaboration in a multi-laboratory preclinical study

**DOI:** 10.1371/journal.pone.0273077

**Published:** 2022-08-25

**Authors:** Jenna M. Evans, Alexander Peever, Agnes Grudniewicz, Braedon McDonald, Asher A. Mendelson, Manoj M. Lalu

**Affiliations:** 1 DeGroote School of Business, McMaster University, Hamilton, Canada; 2 Telfer School of Management, University of Ottawa, Ottawa, Canada; 3 Department of Critical Care Medicine, Snyder Institute for Chronic Diseases, Cumming School of Medicine, University of Calgary, Calgary, Canada; 4 Rady Faculty of Health Sciences, Department of Medicine, Section of Critical Care Medicine, University of Manitoba, Winnipeg, Canada; 5 Department of Anesthesiology and Pain Medicine, University of Ottawa, Clinical Epidemiology and Regenerative Medicine Programs, Ottawa Hospital Research Institute, Ottawa, Canada; Public Library of Science, UNITED STATES

## Abstract

**Introduction:**

Medical advancements are slow to reach the patient bedside due to issues with knowledge translation from preclinical studies. Multi-laboratory preclinical studies are a promising strategy for addressing the methodological deficiencies that weaken the translational impact of single laboratory findings. However, multi-laboratory preclinical studies are rare and difficult, requiring strong collaboration to plan and execute a shared protocol. In multiteam systems such as these, collaboration is enhanced when members have cohesive ways of thinking about their goals and how to achieve them–that is, when they have “shared mental models”. In this research project, we will examine how members of Canada’s first multi-laboratory preclinical study build shared mental models and collaborate in the execution of their study.

**Methods:**

Six independent labs in Canada will conduct a preclinical study using a common protocol. To investigate mental models and collaboration in this multiteam system we will conduct a longitudinal qualitative study involving interviews at four time points, team observation, and document analysis. We will analyze interview transcripts using deductive coding to produce a matrix analysis of mental model content over time and inductive coding to produce a thematic analysis of members’ experiences of collaboration over time. We will also triangulate data sources to “tell the story” of teamwork, capturing events and contextual information that explain changes in mental models and collaboration over time.

**Discussion:**

This study will be one of the most comprehensive longitudinal analyses of a real-world multiteam system, and the first within a preclinical laboratory setting. The results will contribute to our understanding of collaboration in multiteam systems, an organizational form increasingly used to tackle complex scientific and social problems. The results will also inform the implementation of future multi-laboratory preclinical studies, enhancing the likelihood of effective collaboration and improved ‘bench to bedside’ translation.

## Introduction

Medical advancements are often slowed due to difficulties in translating preclinical and clinical studies to the patient bedside. Multi-laboratory preclinical studies are a promising new strategy for addressing the methodological deficiencies that weaken the translational impact of single laboratory studies [1,2]. These types of studies involve two or more research labs collaborating on study design, execution, and analysis using a shared protocol [3]. Through this collaborative approach, multi-laboratory preclinical studies increase transparency, improve reproducibility, and enhance generalizability. Although multicenter studies are considered the gold standard in clinical research, they are rare in preclinical research [4]. Multi-laboratory studies are time-consuming and difficult, requiring strong interdisciplinary collaboration to develop and execute a consistent protocol, manage issues, and share results [3,5]. The present research examines the process of collaboration in Canada’s first multi-laboratory preclinical study which brings six labs together to develop a validated and replicable sepsis model.

Multi-laboratory preclinical studies involve a variety of stakeholders, including basic scientists, clinical scientists, research technicians, trainees, and lab animal veterinarians, working across geographically distributed labs. In a “publish or perish” academic culture characterized by hierarchy, competition, and first-mover advantage, individuals engaged in preclinical research are not only unaccustomed to collaborating but are often resistant to doing so [6]. Furthermore, labs are embedded in institution-specific contexts with distinct pressures, resources, logistical processes, and behavior norms that reinforce divisions [3,4]. Although major funders are beginning to invest in multi-laboratory studies [7], there are currently no roadmaps for successful implementation. A better understanding of these highly structured collaborations may also provide insight into less formal types of collaboration that occur more frequently between labs.

Given the barriers to collaboration in multi-laboratory preclinical studies, interdisciplinary research that leverages the rich theoretical and empirical literature on teams could prove useful [8]. Teams research is a domain of the broader discipline of organizational science that focuses on team functioning and effectiveness within and across organizations. The ‘science of team science’ is a parallel domain explicitly focused on collaborative team-based approaches to scientific research [9]. A teams lens can be used to explicate the process and dynamics of collaboration to establish a roadmap for future multi-laboratory preclinical studies. Drawing from teams research, a multi-laboratory preclinical study can be conceptualized as a “multiteam system”, defined as two or more teams that interface directly and interdependently toward the accomplishment of collective goals [10–13]. Multiteam systems are larger and structurally more complex than single teams with members spanning distinct professional groups, teams, organizations, and geographic locations. As a result, it is particularly challenging for members to find “common ground” and get “on the same page” to accomplish superordinate goals [14–16]. Members often have separate and disjointed ways of thinking about system goals and the means to achieve them–that is, they have divergent “mental models”. Mental models are internal psychological representations of external reality [17,18]. In other words, they are small-scale models in the mind of how the world works which enable individuals to interpret situations and take action [17]. Mental models develop over time through experiences, interactions with others, and vicarious learning [19]. A lack of shared mental models among multiteam system members hampers their ability to collaborate and coordinate their efforts [14,20–22].

To date, no longitudinal studies of teamwork have been conducted in preclinical research settings. We therefore know little about how members of multi-laboratory preclinical research teams “get on the same page” (or fail to) in the execution of their studies. The application of a teams lens to a multi-laboratory preclinical study may offer new theoretical and practical insights for both preclinical/translational medicine and organizational science.

## Research objectives

We will conduct a longitudinal qualitative study of Canada’s first multi-laboratory preclinical study as team members plan and execute a common experimental protocol. Our research question is, how do collaboration and shared mental models evolve over time in a multi-laboratory preclinical study? Specific research objectives include to: (a) Describe how the multiteam system collaborates over time (or fails to), (b) Identify where members’ mental models converge and diverge over time, and (c) Integrate findings from objectives (a) and (b) to develop a theoretical model and recommendations to inform future research and practice.

## Theory

Shared Mental Model Theory provides a framework for examining the extent to which members of the multi-laboratory preclinical study are “on the same page”. When team members develop a common psychological understanding of their work, this is referred to as a shared mental model [23,24]. There are two types of shared mental models. Shared *task* mental models refer to a shared understanding of team goals, sequence of activities, and contingency plans [23]. Shared *team* mental models refer to a shared understanding of team member roles and interaction patterns [23]. Reviews spanning 15 years confirm shared mental models positively predict team functioning and performance [24–26]. Shared mental models permit a co-understanding of what is happening, what is likely to happen next, and why it is happening, thus enabling coordinated and collaborative behaviors in the completion of interdependent tasks, even in the absence of direct communication [23,24,27,28]. For example, in a healthcare study, higher performing simulated trauma teams demonstrated more behaviors indicative of shared mental models than lower performing teams [29]. Similarly, in a field study of air traffic control tower teams, the highest efficiency and safety rates were evident only when air traffic controllers exhibited both shared task and team mental models [30].

Our application of shared mental model theory to a multiteam system conducting preclinical research using longitudinal qualitative methods is novel for five reasons. First, shared mental models have been identified as a “critical lever” for multiteam system functioning and effectiveness [11,12,22]. However, most studies examine shared mental models in single teams, not across multiteam systems [11]. Second, studies of shared mental models tend to focus on military, air traffic control, business, and healthcare teams, particularly trauma and surgical teams [24,31]. The theory has not been applied in the context of preclinical, lab-based teamwork. Third, most studies of shared mental models are conducted on simulated teams, not real-world teams [24,32]. Fourth, most studies measure shared mental models at one point in time rather than longitudinally over time [33]. Studies of multiteam systems also tend to involve data collection at a single point in time [11,34]. Finally, quantitative methods dominate in the literature on shared mental models, such as the use of network analysis algorithms to compare members’ pairwise comparison ratings or concept maps [24,35]. These quantitative methods are appropriate for studying teamwork involving immediate, predictable action as found in control crew, military, or surgical teams, but less appropriate in complex virtual teams undertaking more ambiguous tasks–such as in a geographically distributed multi-laboratory preclinical research team. Qualitative methods can provide a more comprehensive picture of the context and dynamics of how shared mental models evolve over time in a preclinical multi-laboratory study.

## Methods

We will conduct a longitudinal qualitative study involving interviews, team observation, and document analysis over 18–24 months between 2021 and 2023. These qualitative methods will generate rich, contextualized data grounded in team member experiences and conducive to building theory and informing practice [8,36]. Our focus is on delineating the process by which shared mental models and collaboration emerge, evolve, or decline in the multi-laboratory preclinical study, and on revealing the conditions that underlie shifts over time [37].

### Study setting

The National Preclinical Sepsis Platform (NPSP) is a scientific consortium undertaking Canada’s first multi-laboratory preclinical study, as part of the Sepsis Canada Research Network funded by the Canadian Institutes for Health Research. The NPSP brings together six independent labs from three Canadian provinces (Ontario, Alberta, and Manitoba) to collaboratively conduct an experiment using a shared protocol [2]. The objective of their first study is to explore the influence of biological sex on host response and treatment effect from fluid resuscitation and antibiotics using a fecal-induced peritonitis (FIP) model of murine sepsis. Although sex-dependent influences have been demonstrated in various medical conditions, it is currently unclear how biological sex may affect outcomes in sepsis [38,39]. Moreover, for intravenous fluids and antibiotics (the two cornerstones of sepsis therapy) there is a lack of robust and reliable data evaluating how biological sex influences the effectiveness of these treatments [40].

Pilot studies will be designed by the full group and executed at individual labs in order to optimize particular aspects of the experimental protocol, such as dose-response disease severity of the fecal slurry, timing of fluids and antibiotics, and operational feasibility of the model. Then, the full study will be undertaken simultaneously at all labs across Canada. Regular meetings occur every 4–6 weeks involving all core labs, including principal investigators, highly-qualified personnel (i.e. technicians), and graduate students.

Four individuals with lived experience of sepsis either as a patient or caregiver are “patient partners” with the NPSP. Patient engagement in preclinical research is rare and challenging [41]. Laboratory-based scientists do not typically interact with patients, unlike clinical scientists. The NPSP’s objective in engaging patients is to align the platform’s work with outcomes that matter to patients. The patient partners attend meetings to gain an understanding of the research and to provide input on priorities, outcome measures, interpretation of findings, and accessible communication based on their experiences. Although patients are involved in the preclinical study itself, they are not involved in the presently described study of collaboration. However, if early data suggest that patient engagement is a key process in or influencing factor on inter-lab collaboration, we will take the necessary steps to gather additional data from team members and patient partners.

### Data collection

**Fig 1:**
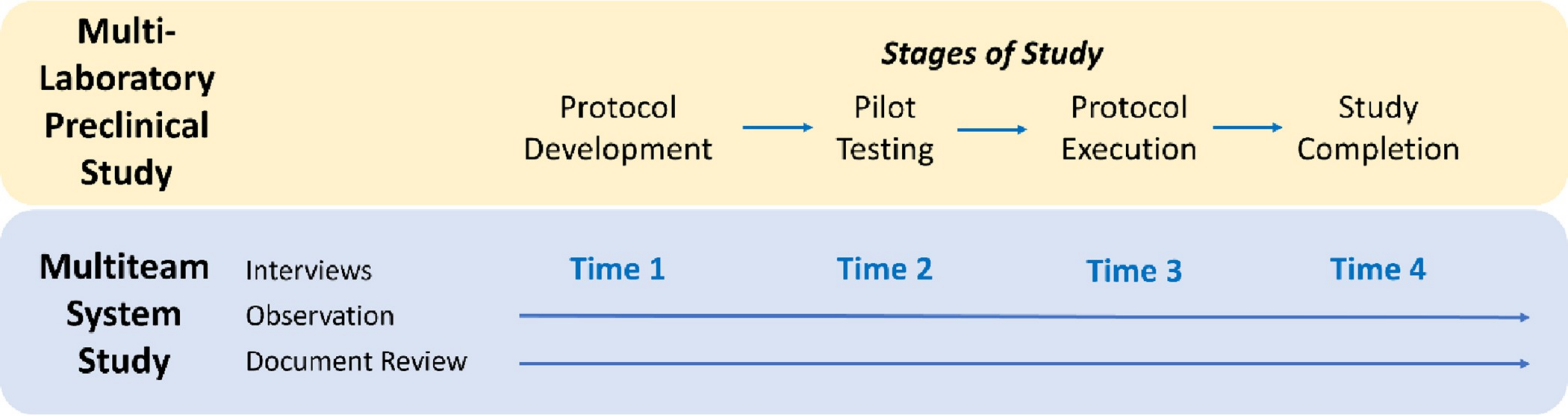
Longitudinal data collection plan.

Our primary method of data collection is semi-structured interviews at four time points to elicit mental model content and team member experiences of collaboration. Each lab consists of approximately 3–6 core members including investigators, veterinarians, research technicians, and graduate students for a total of approximately 30 members. All members will be invited via email to participate in four 60-minute semi-structured individual interviews, the first in the early stages of protocol development, the second during pilot testing, the third immediately after protocol execution, and the fourth after study completion for a total of approximately 120 interviews (Fig 1). Participant names and e-mail addresses will be obtained through the NPSP. Interviews will be conducted virtually due to geographical dispersion, digitally recorded, and transcribed verbatim by a professional transcriptionist.

The interview guides are semi-structured, allowing for comparison over time and across participants as well as probing on unexpected topics and events [42]. Four types of theory-driven, open-ended interview questions will be asked to elicit data on participants’ mental models and experiences of collaboration over time. First, we will ask what is going well in the multi-laboratory study and what is not going well. These two broad questions allow participants to describe what is most salient to them in their own words. Second, we will ask questions that elicit participants’ task mental models, such as: What are the goals of this multi-laboratory study? What is required to achieve these goals? What are the key phases or tasks involved in executing this study? Third, we will ask questions that elicit participants’ team mental models, such as: What is the role of each lab/member? How do you keep in touch with individuals at other labs? To what extent and how are individuals from the different labs working together as a team? What is needed to improve collaboration across labs? Finally, we will prompt participants to identify and describe “critical incidents”, such as the emergence of new information, changes in the environment, challenges, errors, or conflicts that influenced the team’s work and interactions.

Interview data will be supplemented by secondary sources, including relevant documents (e.g., protocols, meeting minutes), team emails, brief informal monthly interviews with select members, as needed, and field notes from observation of monthly team meetings. This data will provide contextual information and insight into team activities and interactions which can be used to triangulate and extend the formal interview data.

This study has ethics approval from the Hamilton Integrated Research Ethics Board (13571). NPSP members received an information sheet about the research and a consent form. The information sheet included details on the voluntary nature of the study and how confidentiality would be maintained throughout the research process. To provide consent for meeting observation, participants signed a written consent form. Notes taken during observation are anonymized and focused on content related to study execution and on group dynamics, not on individuals. Prior to an interview, information from the consent form is reiterated verbally and participants are asked to consent to participation and recording of their interviews. Transcripts are labelled with participant ID codes and stored separately from names and other identifying information. For the preclinical component of this work, each NPSP lab obtained ethics approval from their local animal ethics committee. Members of the local animal care committees were consulted to establish commonly accepted practices for experimental design across all labs.

### Data analysis

Drawing from the multiple data sources, we will develop a narrative and associated timeline to “tell the story” of teamwork in the multi-laboratory preclinical study, capturing key events and contextual information that may help explain changes over time in collaboration and shared mental models [37]. To minimize potential bias, all methodological decisions will be documented in an audit trail, members of the qualitative analysis team will independently code portions of the data and reach consensus through discussion, and ‘member checking’ with participants will be conducted at multiple points during the study to improve the accuracy of interpretations [42].

We will analyze interview transcripts using hybrid deductive and inductive coding in QSR International’s NVivo software. Coding is a process of systematically labeling and organizing qualitative data extracts to identify themes and relationships between them [42]. Deductive coding involves the use of a predefined, theory-based set of codes, while inductive coding involves creating codes based on the data itself. Both coding approaches are necessary to answer our research questions. We will use deductive coding based on shared mental model theory to analyze the team’s mental models and inductive coding to analyze team member experiences of collaboration. We will deductively code the transcripts using key constructs from shared mental model theory, including ‘task mental models’ with sub-codes such as ‘goals’ and ‘team mental models” with sub-codes such as ‘roles’. We will use a matrix template to systematically extract and compare mental models across team members [43]. The matrix will consist of rows (team members), columns (codes), and cells of summarized data, providing a structure into which we will systematically reduce the data in order to analyze it by team member and by code (i.e., mental model content area). Cells will summarize the key concepts and associated statements (two or more concepts and their relation) evoked by each team member for each code; this content constitutes their ‘mental model’ [44,45]. Deductive coding and the matrix output will enable us to determine mental model content and similarity across team members and labs over time. Concepts and statements that are common in at least 50% of members in any given unit (e.g., component team, professional group, multiteam system) will be considered ‘shared’ [45]. Use of a matrix template as an analytical tool for systematically tracking and comparing mental models is novel and may help advance qualitative measurement of shared mental models.

We will simultaneously inductively code the transcripts to analyze team member experiences of collaboration and critical incidents that may reinforce or challenge shared mental models and collaboration. Through line-by-line analysis we will identify common statements and group them into categories known as ‘first order’ codes [42]. Then we will search for relationships and/or variations among these first-order codes and further consolidate them into categories known as ‘second-order’ codes that are more abstract and theoretical [42].

In the final stage of analysis, we will draw from the results of our matrix and inductive analyses to develop a theoretical model that reflects patterns identified in the data on how mental models and collaboration evolved over time and the mechanisms underlying those patterns [37]. Once the model is finalized, we will review the data to look for confirming and disconfirming evidence for the model and to ensure that no relevant codes were missed. In addition to explicating how mental models and collaboration evolved over time, the theoretical model will help identify propositions and hypotheses to guide future research as well as practical recommendations to inform future multi-laboratory preclinical studies. The recommendations will be based on multiple stakeholder perspectives and experiences, and will focus on how these teams can build shared mental models, collaborate effectively at different stages of their study, and avoid common pitfalls.

## Discussion

A defining feature of this study is the use of longitudinal qualitative methods to elucidate *how* shared mental models and collaboration evolve over time. While this approach addresses gaps in the literature and has strong potential to generate new knowledge, it is very time-consuming and resource intensive. The study involves following the multiteam system for an extended period of time (18–24 months) and will generate a large qualitative data set consisting of hundreds of pages of data that require careful processing and analysis at multiple time points. Furthermore, while matrices have been used broadly in qualitative research to organize and reduce data for systematic comparison [43], this method has not been applied to the measurement of shared mental models. The challenges associated with building and managing a large qualitative data set with novel components will be mitigated by employing a strong team of qualitative researchers and coders who meet regularly to problem-solve and ensure consistency in data collection and analysis.

This study will occur in a unique context characterized by geographic and cultural differences across Canada as well as the COVID-19 pandemic. Participating labs are spread across three provinces, Ontario, Manitoba, and Alberta, each with distinct social, economic, and political profiles that may influence team dynamics. Furthermore, geographic dispersion requires that teams use virtual meeting technology as the primary mode of communication. With the onset of the pandemic, these technologies were streamlined and quickly became mainstream. In an “era of zoom”, the potential for geographically dispersed multiteam systems, such as this, to function smoothly and productively is greatly enhanced. This multiteam system may also be influenced by the oscillation between lockdowns and re-openings due to the pandemic, as labs are forced to shut down and restart activities to comply with local legislation. Our qualitative research design incorporates consideration for contextual factors such as these in our analyses and heeds a call in the literature for research to “pay more attention to where multiteam systems “live and operate” [36].

## Conclusion

Our proposed study will examine how members of Canada’s first multi-laboratory preclinical study work together across professional, organizational, and geographic boundaries to execute a shared experiment. This interdisciplinary study will advance knowledge in both the organizational and biomedical sciences. The results will contribute to our understanding of shared mental models and collaboration in a unique multiteam system characterized by geographical distribution and a highly complex task domain. These characteristics enhance the relevance of our study to the increasing use of multiteam systems to tackle global problems such as pandemic response and counter terrorism. Our focus on shared mental models, a critical but understudied property of multiteam systems, will also help inform the development of team interventions that enhance multiteam system effectiveness, such as training, planning exercises, and team charters [11]. Furthermore, use of a matrix template as an analytical tool for systematically tracking and comparing mental models is novel and may help advance qualitative measurement of shared mental models.

In the biomedical sciences, the results will inform the execution of future multi-laboratory preclinical studies. We will develop a roadmap on how to establish multi-laboratory preclinical teams that includes consideration for how the demands of the work evolve over time. A better understanding of multi-laboratory preclinical studies will also provide insight into more informal types of collaboration that frequently occur between labs. The roadmap we develop will enhance the likelihood that individuals develop shared mental models and collaborate as they work towards preclinical discoveries and successful translation of promising findings into human studies.
